# Effect of Vinblastine Timing on Skin Graft Healing in a Rat Model

**DOI:** 10.3390/medicina62040624

**Published:** 2026-03-25

**Authors:** Eren Tuncer, Elif Gündeş Alten, Aytaç Alten, Erol Kozanoğlu, Şule Öztürk Sarı, Ufuk Emekli

**Affiliations:** 1 Department of Plastic, Reconstructive and Aesthetic Surgery, Basaksehir Cam and Sakura City Hospital, 34480 Istanbul, Türkiye; erentuncr@gmail.com; 2 Department of Plastic, Reconstructive and Aesthetic Surgery, Istanbul Faculty of Medicine, Istanbul University, 34116 Istanbul, Türkiye; erol.kozanoglu@istanbul.edu.tr (E.K.); ufukemekli@yahoo.com (U.E.); 3 Department of Plastic, Reconstructive and Aesthetic Surgery, Gaziosmanpasa Research and Training Hospital, 34255 Istanbul, Türkiye; altenayt@gmail.com; 4 Department of Medical Pathology, Istanbul Faculty of Medicine, Istanbul University, 34116 Istanbul, Türkiye; suleozturksari@gmail.com

**Keywords:** vinblastine, skin graft, wound healing, timing, chemotherapy, angiogenesis

## Abstract

*Background and Objectives*: Chemotherapeutic agents are known to disrupt wound healing; however, the influence of administration timing on skin graft repair remains insufficiently characterized. This study aimed to investigate the time-dependent effects of vinblastine exposure on full-thickness skin graft healing in a rat model. *Materials and Methods*: Twenty-four female Wistar albino rats were allocated into four groups (*n* = 6). The control group underwent grafting without pharmacologic intervention, whereas the experimental groups received a single intraperitoneal dose of vinblastine (2 mg/kg), followed by grafting in the first week, second week and third week after administration. Graft specimens were harvested on postoperative day 7 for histopathological evaluation performed by a blinded pathologist. Hematoxylin-eosin-stained sections were scored for inflammation, granulation tissue formation, fibroblast maturation, collagen deposition, re-epithelialization, and neovascularization. Intergroup comparisons were conducted using the Kruskal–Wallis test with Dunn–Bonferroni post hoc analysis. *Results*: Vinblastine exposure produced significant time-dependent differences in several healing parameters. Fibroblast maturation was markedly reduced in the second-week graft group compared with controls (*p* < 0.001). Re-epithelialization was significantly delayed in the second- and third-week groups (*p* = 0.033). Granulation tissue formation differed between groups (*p* = 0.014), with higher early scores observed in the first-week group. Notably, neovascularization was significantly greater in the third-week group than in the control and second-week groups (*p* = 0.010), suggesting partial recovery of angiogenic activity over time. No significant differences were detected in inflammation or collagen deposition. *Conclusions*: Vinblastine exposure appears to exert time-dependent effects on skin graft healing, with the second week representing a period of less favorable histopathological repair. Partial recovery observed with later grafting suggests that the interval between chemotherapeutic exposure and reconstructive procedures may influence graft outcomes and support improved surgical planning.

## 1. Introduction

The graft is tissue that has been completely separated from its original location and has had its blood supply severed, intended for surgical transfer to a suitable recipient site [1]. This tissue may be from the patient themselves (autologous) or from another individual (heterologous) [2]. One of the commonly used graft types in wound reconstruction is the skin graft, which can be classified as full-thickness and partial-thickness skin grafts [3]. The grafts play a critical role in plastic surgery for many indications; they are successfully used in the repair of traumatic tissue loss, burn reconstruction, closure of oncological resection sites, correction of scar contractures, treatment of congenital anomalies, and treatment of some chronic ulcers [4,5,6]. According to the reconstructive ladder approach, grafting is one of the preferred alternatives after secondary healing and primary repair [7]. Skin grafting is one of the fundamental techniques in plastic surgery today, due to its minimally invasive nature and wide range of indications [8].

The adherence of the skin graft in the area where it is transferred depends on the interaction of numerous systemic and local factors. Surgical parameters such as the graft’s surface area, thickness, method of removal, and the level of vascularization in the area where it is placed are important factors that directly affect the graft’s oxygenation and nutrition and determine its survival [9]. At the same time, complications such as infection, hematoma, or seroma at the recipient site can negatively affect graft healing [10]. In addition, factors such as the patient’s age, nutritional status, systemic diseases, and smoking also directly affect wound healing [10]. Molecular processes such as the balance of the inflammatory response, fibroblast maturation, and collagen synthesis during wound healing are also influential [11]. Based on this, when examining graft survival, it is seen that molecular processes play a role alongside surgical techniques.

In the current literature, the adverse effects of chemotherapeutic agents on wound healing are demonstrated. These drugs prolong inflammation, suppress angiogenesis, and reduce extracellular matrix production by inhibiting the proliferation of fibroblasts, keratinocytes, and endothelial cells [11]. In experimental models undergoing chemotherapy, this situation leads to delayed wound closure, chronic wound formation, and impaired tissue regeneration [11,12]. Studies involving clinical cases have also reported prolonged graft healing times, increased infection rates, and increased need for revision during and after surgical interventions following chemotherapy [13]. Vinblastine, one of the chemotherapeutic agents used in the treatment of hematologic malignancies and certain solid tumors, is a drug belonging to the vinca alkaloids class and acts by affecting microtubules during mitosis [14]. It is known that vinca alkaloids inhibit cell division and induce apoptosis, and they also have various effects on angiogenesis inhibition and inflammation [15]. These pharmacodynamic properties suggest that vinblastine may affect graft survival. It is known that the timing of administration of chemotherapeutic agents affects wound healing [16]. This suggests that administering vinblastine at different time intervals prior to graft surgery may influence the inflammatory response and tissue regeneration process. However, specific experimental evidence regarding the effect of vinblastine timing on full-thickness skin graft healing remains limited. Therefore, this study aimed to evaluate the effects of vinblastine on graft healing using different timing protocols and to assess its impact on histopathological wound repair dynamics.

## 2. Materials and Methods

This study was approved by the Istanbul University Local Animal Experiment Ethics Committee (No. 2022/01, 28 January 2022). The sample size was determined based on Görgülü et al. and an a priori power analysis performed using G*Power 3.1 [17,18,19]. Assuming a medium-to-large effect size (f = 0.42), with α = 0.05, power (1 − β) = 0.80, and four experimental groups, the required total sample size was calculated as 24 animals (*n* = 6 per group), confirming the adequacy of the study design. A total of four groups were formed in the study, and a specific experimental protocol was applied to each group.

Twenty-four female Wistar albino rats (8–10 weeks, 250–300 g) were randomly allocated into four equal groups (*n* = 6) using a pre-generated computer-based random sequence. Allocation was performed by an investigator not involved in surgery or histopathologic evaluation. Histopathologic assessments were performed by one blinded pathologist.

Group 1 (control group) underwent full-thickness skin graft surgery without the administration of any pharmacological agents. Groups 2, 3, and 4 received a single intraperitoneal dose of 2 mg/kg vinblastine (Vinko®, Koçak Farma, Tekirdağ, Türkiye) at different intervals prior to grafting (1, 2, and 3 weeks before surgery, respectively). All animals underwent graft surgery on the same operative day under identical conditions. This protocol allowed evaluation of the temporal effects of vinblastine exposure on graft healing. Prior to graft surgery, a combination of 50 mg/kg ketamine hydrochloride and 7 mg/kg xylazine hydrochloride was administered intraperitoneally to provide general anesthesia to the rats. Body temperature, measured rectally, was maintained at approximately 37 °C; 20 mg/kg cefazolin was administered intramuscularly for prophylaxis prior to surgery. During the surgical procedure, a 2.5 × 2.5 cm square skin island was marked on the interscapular region of the rats. A full-thickness skin graft, including the epidermis, dermis, and subcutaneous tissue, was obtained from this area using sharp dissection. The excised graft was carefully examined ex vivo and trimmed to uniform thickness, particularly in terms of thickness. This is important for both the complete adaptation of the graft to the recipient site and the healthy initiation of the revascularization process [20,21]. Once hemostasis was achieved, the same graft tissue was anatomically repositioned in the area from which it was taken and secured by suturing the edges with 4/0 monofilament suture material. A classic bandage-type dressing was applied over the graft to prevent movement and surface fluid accumulation. This technique is widely preferred in full-thickness graft models to increase graft retention and mechanical stability in the early period [22].

Seven days after graft surgery in all groups, rats were sacrificed under high-dose anesthesia (200 mg/kg ketamine + 20 mg/kg xylazine, intraperitoneal). Following sacrifice, the graft areas were excised, including the deep fascia, and placed in appropriate fixatives for histopathological examination. Histopathological evaluation was performed on hematoxylin–eosin (H&E)-stained sections. Seven parameters were assessed: acute inflammation, chronic inflammation, amount of granulation tissue, granulation and fibroblast maturation, collagen formation, re-epithelialization, and neovascularization. All evaluations were performed by a single blinded pathologist. For each animal, one mid-graft section from the graft site was selected for analysis. Five non-overlapping high-power fields (HPFs) per specimen were systematically evaluated at ×200 magnification, including predefined central and peripheral areas of the graft to ensure representative sampling. Each parameter was scored as 0, 1, 2, or 3 according to the scoring system defined by Abramov et al. [17,23] (Table 1). A single value per animal was calculated as the mean of the five HPF scores.

All statistical analyses were performed using IBM SPSS Statistics software (version 27.0, IBM Corp., Armonk, NY, USA). Although histopathological parameters were scored on an ordinal 0–3 scale, each value represents the average of multiple high-power field evaluations per specimen and is therefore presented descriptively as mean ± standard deviation. Due to the ordinal origin and non-normal distribution of the data, intergroup comparisons were conducted using the Kruskal–Wallis test. When significant, pairwise comparisons were performed using the Dunn–Bonferroni post hoc test. A *p*-value < 0.05 was considered statistically significant.

## 3. Results

In the study, data obtained from histopathological evaluations of graft sites in four different groups were analyzed, and parameters showing significant differences were determined and presented in Table 2. No treatment-related adverse effects, unexpected deaths, infections or systemic toxicity findings were observed during the study period.

Significant intergroup differences were detected for granulation tissue amount (*p* = 0.0149), granulation and fibroblast maturation (*p* = 0.0007), re-epithelization (*p* = 0.0337), and neovascularization (*p* = 0.0105). No statistically significant differences were observed for acute inflammation, chronic inflammation, or collagen deposition (*p* > 0.05).

Pairwise comparisons (Table 3) showed higher granulation tissue scores in Week 1 and Week 3 compared with the Control group. For granulation and fibroblast maturation, the Control group showed significantly higher scores than all experimental groups, while Week 2 demonstrated significantly lower scores than Week 1 and Week 3. Re-epithelialization scores were lower in Week 2 compared with Week 1 and the Control group. Neovascularization was significantly higher in Week 3 compared with Week 2 and the Control group.

**Figure 1:**
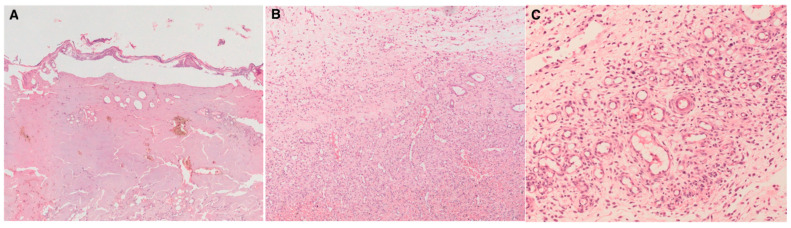
Representative histological findings from graft sites in different experimental groups. (**A**) Control group showing complete but relatively thin re-epithelialization on the epidermal surface, with regularly organized granulation tissue and fibroblasts in the superficial dermis. (**B**) Week 2 graft group demonstrating less organized granulation tissue with reduced fibroblast maturation and persistent inflammatory cell infiltration within the dermal layer. (**C**) Week 3 graft group showing prominent neovascularization characterized by numerous newly formed capillary structures within the granulation tissue. Hematoxylin–eosin staining; original magnification ×40 (**A**) and ×200 (**B**,**C**).

Representative histological findings corresponding to the observed differences between groups are shown in Figure 1.

## 4. Discussion

Skin graft survival relies on a tightly coordinated healing process involving inflammation, cellular proliferation, and angiogenesis [4]. While the adverse effects of chemotherapeutic agents on wound healing are well recognized, the impact of administration timing remains insufficiently understood [12]. The present study suggests that vinblastine exposure is associated with time-dependent alterations in early graft healing, with less favorable histopathological findings observed when grafting was performed during the second week after administration. These results indicate that timing may influence early biological responses in graft repair.

Significant intergroup differences were observed in granulation tissue formation and fibroblast maturation, both of which are key indicators of effective wound repair [23]. The lowest maturation scores were detected in the second-week graft group, whereas the control group demonstrated more advanced fibroblast organization. This pattern may reflect transient suppression of fibroblast activity following vinblastine exposure, potentially impairing matrix organization and structural repair. Given the central role of fibroblasts in graft integration, this temporal pattern may reflect transient biological susceptibility during early repair. These observations are consistent with the known microtubule-inhibitory effects of vinblastine on proliferating cells [24].

Granulation tissue formation is a key marker of early reparative activity in graft healing [23]. In the present study, significantly higher granulation scores were observed in the first-week graft group following vinblastine administration. This finding may indicate that the early reparative response remains relatively preserved shortly after exposure, whereas the lower granulation scores detected in the second week suggest a delayed inhibitory effect on fibrovascular proliferation. This temporal pattern supports the possibility that the biological impact of vinblastine on wound healing may evolve over time rather than occur immediately after exposure.

Significant intergroup differences were also identified in re-epithelialization. The second-week graft group demonstrated markedly lower epithelial scores compared with controls, suggesting transient suppression of epidermal regenerative capacity following vinblastine exposure. This observation is consistent with the established inhibitory effects of microtubule-targeting chemotherapeutic agents on keratinocyte proliferation and migration [11].

Neovascularization findings further supported a time-dependent healing response. The third-week graft group exhibited significantly greater vascular proliferation than both the control and second-week groups, indicating partial recovery of angiogenic activity over time. This may suggest partial recovery of angiogenic activity over time following vinblastine exposure. These findings align with previous evidence demonstrating the antiangiogenic effects of chemotherapeutic agents on endothelial cell function [25].

Evidence regarding the effects of vinca alkaloids on wound healing remains limited. Although extracts derived from Catharanthus roseus have been associated with enhanced repair, vinblastine is an antimitotic agent with distinct cytotoxic properties [26]. Previous studies focusing on microvascular anastomosis reported no significant impairment in vascular healing [24,27]. In contrast, our findings demonstrate a time-dependent inhibitory effect on graft healing, particularly during the second week after exposure. This discrepancy may reflect differences in tissue biology, as graft repair requires coordinated fibroblast activity, epithelial regeneration, and matrix remodeling beyond vascular patency alone. These findings should also be interpreted within the broader experimental literature demonstrating that chemotherapeutic agents may impair wound healing by inhibiting cellular proliferation, angiogenesis, and extracellular matrix remodeling processes [11].

### Limitations

This study has several limitations. First, vinblastine was administered as a single dose; therefore, potential dose-dependent effects were not evaluated. Second, histopathological assessment was limited to light microscopy, and no immunohistochemical, molecular or objective histomorphometric analyses were performed, which may have provided additional insight into the mechanisms underlying the observed healing patterns. All scoring was performed by a single blinded pathologist, and interobserver reproducibility was not assessed. And histopathological assessment was performed at a single postoperative time point and did not include functional outcomes such as graft take percentage, necrosis area, tensile strength, or perfusion measurements. Therefore, the findings should be interpreted within the scope of early histological changes. Additionally, a full-thickness skin graft model was used; therefore, extrapolation of these findings to split-thickness grafts should be made with caution. Finally, as this was an experimental animal study, caution is warranted when extrapolating these findings directly to clinical practice. Nevertheless, controlled experimental models remain valuable for isolating biological responses and generating hypotheses for future translational research.

## 5. Conclusions

Vinblastine exposure was associated with time-dependent histopathological differences in skin graft healing. Grafting performed during the second week after administration was associated with less favorable histopathological repair, whereas later grafting demonstrated partial recovery of healing responses. These findings suggest that treatment timing may influence early biological aspects of graft repair in this experimental model. Further experimental and clinical investigations are required to determine the clinical relevance of these observations.

## Figures and Tables

**Table 1:** Histological Wound Healing Scoring System.

Parameter	Score 0	Score 1	Score 2	Score 3
Acute inflammation	Absent	Mild	Moderate	Severe
Chronic inflammation	Absent	Mild	Moderate	Severe
Granulation tissue amount	Absent	Mild	Moderate	Severe
Granulation and fibroblast maturation	Immature	Mild maturation	Moderate maturation	Complete maturation
Collagen deposition	Absent	Mild	Moderate	Severe
Re-epithelization	Absent	Partial	Complete but immature or thin	Complete and mature
Neovascularization	Absent	Up to 5 vessels per HPF	6–10 vessels per HPF	More than 10 vessels per HPF

Histopathologic parameters were evaluated semi-quantitatively on a 0–3 scale according to the degree of tissue response. Scores of 0 to 3 represent progressive increases in the severity or maturation of each variable, ranging from absence to complete or severe findings. The evaluation included acute and chronic inflammation, granulation tissue amount, fibroblast maturation, collagen deposition, re-epithelialization, and neovascularization. HPF: High-power field.

**Table 2:** Comparison of Histopathological Parameters by Experimental Groups.

Parameter	Week 1 (Mean ± SD)	Week 1 [Median (IQR)]	Week 2 (Mean ± SD)	Week 2 [Median (IQR)]	Week 3 (Mean ± SD)	Week 3 [Median (IQR)]	Control (Mean ± SD)	Control [Median (IQR)]	*p* Value
Acute inflammation	1.83 ± 0.41	2 (2–2)	2.00 ± 0.00	2 (2–2)	1.67 ± 0.52	2 (1–2)	1.67 ± 0.52	2 (1–2)	0.3035
Chronic inflammation	2.17 ± 0.41	2 (2–2)	1.33 ± 0.52	1 (1–2)	2.17 ± 0.41	2 (2–2)	2.17 ± 0.41	2 (2–2)	0.0553
Granulation tissue amount	2.50 ± 0.55	2.5 (2–3)	2.00 ± 0.00	2 (2–2)	2.17 ± 0.41	2 (2–2.5)	1.50 ± 0.55	1.5 (1–2)	**0.0149**
Granulation and fibroblast maturation	2.00 ± 0.00	2 (2–2)	1.00 ± 0.00	1 (1–1)	2.00 ± 0.63	2 (2–2)	3.00 ± 0.00	3 (3–3)	**0.0007**
Collagen deposition	1.67 ± 0.52	2 (1–2)	1.67 ± 0.52	2 (1–2)	2.00 ± 0.00	2 (2–2)	2.33 ± 0.52	2 (2–3)	0.0662
Re-epithelization	2.00 ± 0.00	2 (2–2)	1.33 ± 0.52	1 (1–2)	1.50 ± 0.55	1.5 (1–2)	2.00 ± 0.00	2 (2–2)	**0.0337**
Neovascularization	2.00 ± 0.63	2 (2–3)	1.33 ± 0.52	1 (1–2)	2.50 ± 0.55	2.5 (2–3)	1.50 ± 0.55	1.5 (1–2)	**0.0105**

Granulation tissue formation was highest in the first-week group, while fibroblast maturation and re-epithelialization were lowest in the second week, indicating transient suppression of proliferative activity. The third-week group showed increased neovascularization, suggesting partial recovery of vascular response. No significant differences were observed in inflammatory or collagen deposition parameters (*p* > 0.05). Values are presented as mean ± standard deviation and median (interquartile range). Non-parametric tests (Kruskal–Wallis with Dunn–Bonferroni post hoc analysis) were used for statistical inference. *p* < 0.05 was considered statistically significant.

**Table 3:** Pairwise Comparisons Between Groups.

Parameter	Comparison	Adjusted *p*-Value
**Granulation tissue amount**	Week 1 vs. Control	<0.05
	Week 3 vs. Control	<0.05
**Granulation and fibroblast maturation**	Week 2 vs. Control	<0.001
	Week 1 vs. Control	<0.01
	Week 3 vs. Control	<0.05
	Week 2 vs. Week 1	<0.05
	Week 2 vs. Week 3	<0.05
**Re-epithelization**	Week 2 vs. Week 1	<0.05
	Week 2 vs. Control	<0.05
**Neovascularization**	Week 3 vs. Week 2	<0.05
	Week 3 vs. Control	<0.05

Only statistically significant pairwise comparisons are presented. Adjusted *p*-values were calculated following the overall intergroup analysis.

## Data Availability

The datasets generated and/or analyzed during the current study are available from the corresponding author upon reasonable request.
